# Modulation of resting-state networks following repetitive transcranial alternating current stimulation of the dorsolateral prefrontal cortex

**DOI:** 10.1007/s00429-023-02667-2

**Published:** 2023-07-12

**Authors:** Ahsan Khan, Jochen A. Mosbacher, Stephan E. Vogel, Mira Binder, Michael Wehovz, Arnulf Moshammer, Stefan Halverscheid, Kolja Pustelnik, Michael A. Nitsche, Raymond Kai-Yu Tong, Roland H. Grabner

**Affiliations:** 1 Educational Neuroscience, Institute of Psychology, University of Graz, Graz, Austria; 2 Biomedical Engineering Department, The Chinese University of Hong Kong, Hong Kong, China; 3 Mathematics Institute, University of Göttingen, Göttingen, Germany; 4 Department of Psychology and Neurosciences, Leibniz Research Centre for Working Environment and Human Factors, Dortmund, Germany; 5 Protestant Hospital of Bethel Foundation, Bielefeld University, University Hospital OWL, University Clinic of Psychiatry and Psychotherapy and University Clinic of Child and Adolescent Psychiatry and Psychotherapy, Bielefeld, Germany

**Keywords:** Transcranial alternating current stimulation (tACS), Dorsolateral prefrontal cortex (DLPFC), Functional magnetic resonance imaging (fMRI), Functional connectivity, Frontoparietal network

## Abstract

Transcranial alternating current stimulation (tACS) offers a unique method to temporarily manipulate the activity of the stimulated brain region in a frequency-dependent manner. However, it is not clear if repetitive modulation of ongoing oscillatory activity with tACS over multiple days can induce changes in grey matter resting-state functional connectivity and white matter structural integrity. The current study addresses this question by applying multiple-session theta band stimulation on the left dorsolateral prefrontal cortex (L-DLPFC) during arithmetic training. Fifty healthy participants (25 males and 25 females) were randomly assigned to the experimental and sham groups, half of the participants received individually adjusted theta band tACS, and half received sham stimulation. Resting-state functional magnetic resonance (rs-fMRI) and diffusion-weighted imaging (DWI) data were collected before and after 3 days of tACS-supported procedural learning training. Resting-state network analysis showed a significant increase in connectivity for the frontoparietal network (FPN) with the precuneus cortex. Seed-based analysis with a seed defined at the primary stimulation site showed an increase in connectivity with the precuneus cortex, posterior cingulate cortex (PCC), and lateral occipital cortex. There were no effects on the structural integrity of white matter tracts as measured by fractional anisotropy, and on behavioral measures. In conclusion, the study suggests that multi-session task-associated tACS can produce significant changes in resting-state functional connectivity; however, changes in functional connectivity do not necessarily translate to changes in white matter structure or behavioral performance.

## Introduction

Neuroimaging studies have provided convincing evidence that frequencies generated in the brain are not just a cumulative sum of underlying neural activity but rather represent fundamental mechanisms that drive various functions in the brain (Sejnowski and Paulsen 2006). Non-invasive brain stimulation provides an interesting method to study these brain oscillations and associated behaviors by externally manipulating the target brain region with electric or magnetic currents (Clark and Parasuraman 2014). Transcranial Alternating Current Stimulation (tACS) is of particular interest because it influences cortical excitability in a frequency-dependent manner by aligning the phase of endogenous brain oscillations with externally applied electrical currents (Elyamany et al. 2021). The approach opens an avenue for understanding the causal functioning of the stimulated region and provides a method for possibly enhancing cognitive skills. In numerical cognition, alpha and theta band activity, especially in the left hemisphere, has been associated with different aspects of arithmetic processing (Grabner and De Smedt 2011), and single-session theta band stimulation has been reported to enhance performance in different aspects of arithmetic learning (Hauser et al. 2013; Simonsmeier et al. 2018; Mosbacher et al. 2021). In addition, single-session theta band stimulation over the Left Dorsolateral Prefrontal Cortex (L-DLPFC) has also been reported to induce connectivity changes in resting-state brain networks (Abellaneda-Perez et al. 2019; Mondino et al. 2019). The current study investigated whether multiple-session stimulation of L-DLPFC with theta band stimulation during arithmetic training can induce changes in functional connectivity of resting state grey matter networks and white matter structural integrity.

DLPFC is one of the most crucial brain regions involved in cognitive functions and is often the target in stimulation studies (Dedoncker et al. 2016). From the behavioral point of view, stimulation of the DLPFC has been reported to influence a wide variety of cognitive functions, including attention (Gladwin et al. 2012; Parris et al. 2021), memory (Fregni et al. 2005), arithmetic learning (Hauser et al. 2013; Mosbacher et al. 2021), and various other cognitive domains (Dedoncker et al. 2016). In arithmetic processing, prefrontal regions are an important part of the fronto-parietal network involved in successful task conduction (Grabner et al. 2009; Menon 2015). A recent study systematically comparing single-session effects of alpha and theta tACS targeting the L-DLPFC or the left posterior parietal cortex on arithmetic learning demonstrated that theta tACS of L-DLPFC improves learning of novel arithmetic facts and enhances performance in fact-learning problems (Mosbacher et al. 2021). In addition, a few recent neuroimaging studies have investigated neural changes in relation to one-session theta band stimulation over the DLPFC. For example, Abellaneda-Perez et al. (2019) utilized functional magnetic resonance imaging (fMRI) to investigate the impact of single-session theta band stimulation over the L-DLPFC on resting-state functional networks. The authors of this work reported a significant increase in grey matter functional connectivity in regions of the default mode network (DMN), including the precuneus cortex (PCU), posterior cingulate cortex (PCC), and left inferior parietal lobule (L-IPL). Another tACS study that targeted the L-DLPFC reported a significant increase in functional connectivity between the L-DLPFC and inferior parietal lobule after single-session theta band stimulation over the L-DLPFC (Mondino et al. 2019). These studies demonstrate that a single session of tACS stimulation can manipulate brain activity in brain regions and networks critical for cognitive processing.

Our study builds on these behavioral and neuroimaging findings to investigate whether the effect of multiple-session stimulation along with arithmetic training can induce a significant impact on resting-state brain networks. The DMN, the frontoparietal network (FPN), and the salience network are crucial brain networks involved in cognitive processing (Sridharan et al. 2008). The activity of the DMN decreases whenever a person is focused on anything in the outside world (Greicius et al. 2003). The FPN is a hub of cognitive control, it gets engaged whenever cognitive demand is increased, and mental arithmetic is strongly associated with fronto-parietal activity and connectivity (Greicius et al. 2003; Sridharan et al. 2008; Menon 2015; Marek and Dosenbach 2018), and the salience network acts as a switch between DMN and FPN networks (Goulden et al. 2014). We analyzed these three cognitive networks to investigate if brain stimulation can induce changes in their connectivity patterns.

Besides, numerous research studies utilizing diffusion tensor imaging (DTI) analysis have consistently demonstrated a correlation between performance on arithmetic assessments and measurements of fractional anisotropy (FA) in white matter (WM) pathways that connect the fronto-parietal and cortico-subcortical regions of the brain (Peters and De Smedt 2018; Jeon et al. 2019). Specifically, inferior fronto-occipital fascicle (IFOF), superior longitudinal fasciculus (SLF), and anterior thalamic radiation (ATR) have demonstrated these associations (Peters and De Smedt 2018; Jeon et al. 2019). We further investigated if stimulation can modulate the white matter structural integrity of the tracts commonly associated with arithmetic learning.

In addition, brain structure and function vary greatly between individuals (Forkel et al. 2022), and this variability plays a critical role in determining the impact of stimulation on the targeted region. For instance, Filmer et al. reported that using a priori-defined stimulation protocol can result in individual differences in physiological and behavioral responses to the stimulation (Filmer et al. 2020). In addition, the initial brain state has been demonstrated to play a critical role in determining the impact of tACS (Bullard et al. 2011). Some reports indicate that the alignment of stimulation frequency with the individual dominant frequency determine the efficacy of tACS (Neuling et al. 2013). Despite these findings, most of the existing stimulation studies have not considered individual differences. In our study, we utilized electrophysiological markers (individual alpha frequency [IAF] based on (Klimesch 1999) to individualize the stimulation protocol for each participant to maximize the impact of stimulation on the target region.

In summary, the objective of the study was to understand if multiple sessions of individually adjusted theta band stimulation over the L-DLPFC along with arithmetic training can induce changes in resting-state functional connectivity and white matter structural integrity. During the 5 days of experiment, participants received intensive arithmetic training with personalized theta tACS over the L-DLPFC for 3 days (Day 2 to Day 4). Baseline assessments and post-stimulation assessments were carried out on Day 1 and Day 5. We hypothesized that electrical stimulation would influence resting-state grey matter networks as well as cause modifications in white matter structural integrity.

## Materials and methods

### Study design

**Fig. 1:**
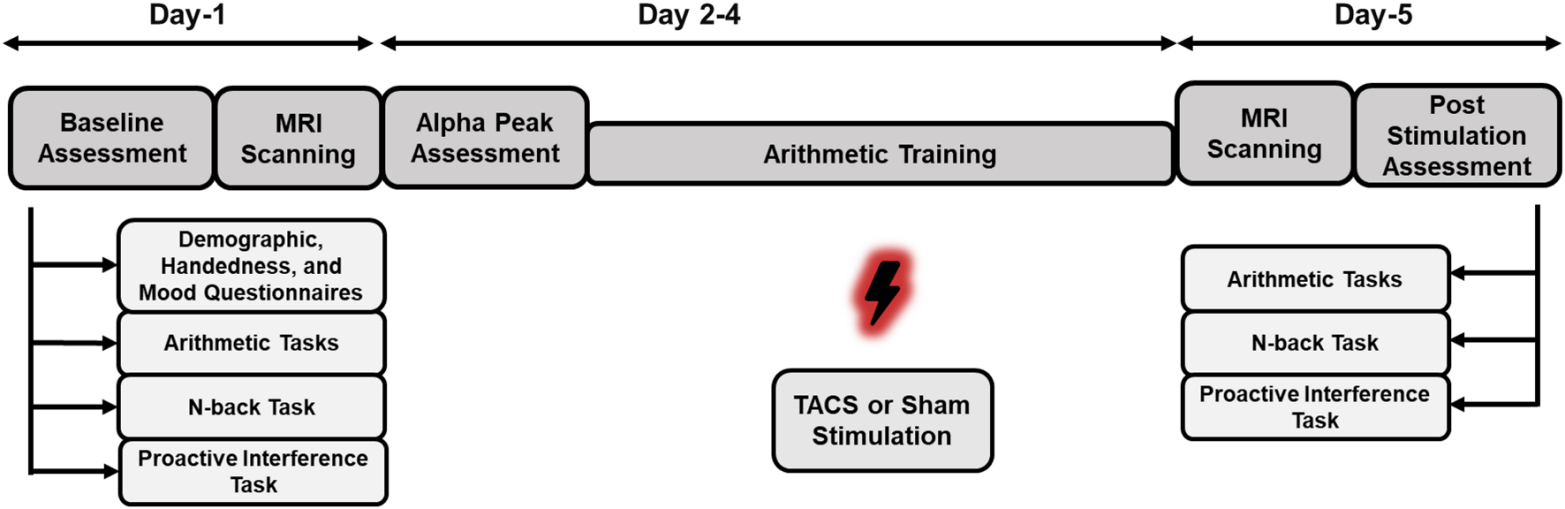
The design of the study. Participants engaged in a 5-day experiment. On day 1, baseline assessments, including demographic, handedness, and mood (MDBF) questionnaires, were filled in. The participant performed computerized working memory (WM; N-Back task) and proactive interference (recent probes task, RPT) tasks. MRI scans were collected after questionnaires and behavioral measures. Alpha peak was assessed from Day 2 to Day 4, and individualized stimulation was administered, during which participants engaged in arithmetic training tasks. On day 5, MRI scanning, arithmetic tasks, N-back task, and proactive interference tasks similar to baseline measures were carried out

The current study was part of a more extensive investigation of the effect of different stimulation training paradigms on arithmetic learning. The current study mainly investigates the functional and structural changes induced by stimulation. The study adopted a between-subjects, double-blinded, placebo-controlled, randomized parallel group design, was in line with the Helsinki declaration on ethical standards and approved by the local ethics committee at the University of Graz. Before any assessments started, the participants were informed about the study goals, and they read the study description. Then they were screened for inclusion and exclusion criteria, and if they were eligible, they were asked to sign a consent to enroll in the study. They were pseudo-randomly assigned to one of the two groups, i.e., experimental and sham. The experiment had a total duration of 5 days. On day one, we collected baseline behavioral and fMRI data. At the start, participants filled out a demographic questionnaire, a questionnaire ascertaining their handedness (Nicholls et al. 2013), and a short mood questionnaire (Steyer et al. 1997). In addition, participants performed two computerized tests assessing working memory (WM; N-Back task) and proactive interference (recent probes task, RPT). Inside the MRI-Scanner, functional data were recorded while participants performed the first blocks of the arithmetic learning task. Additionally, we collected anatomical, Diffusion-weighted imaging (DWI), and resting-state fMRI (rs-fMRI) data in this session. On days 2 to 4, participants came to the electroencephalography (EEG)-Lab, and after a short instruction, the EEG and the tACS electrodes were mounted. On each day, we determined individual stimulation frequency using resting-state EEG recordings and adjusted the stimulation intensity to not cause skin sensations or phosphenes. Participants worked through the arithmetic learning blocks using these individual settings while being stimulated by tACS. On day 5, participants first repeated the WM and proactive interference task outside the scanner. We first collected a second set of anatomical, DWI, and rs-fMRI scans inside the scanner. After these data were recorded, participants worked through the last set of arithmetic learning tasks while we recorded their associated functional brain activity. Finally, they were asked to answer the MDBF, a short questionnaire regarding adverse side effects of tACS, and whether they thought they were truly stimulated or part of the sham group. The design of the study is shown in Fig. 1.

### Participants

Fifty healthy, right-handed subjects (25 females) with normal or corrected-to-normal vision were recruited for this study with a mean age of 23.48 ± 4.13 years. These individuals were randomly divided into a stimulation and sham group. Statistical tests showed no significant group difference in age [*t*(48) = 0.41, *p* = 0.686] and sex [*χ*^2^(1) = 0.08; *p* = 0.777]. We excluded individuals with neurological or psychiatric diseases, neuropsychological or sensory disorders, cardiorespiratory or orthopedic diseases, pregnancy, claustrophobia, MRI contraindications (including implanted metallic or electronic devices), or current regular intake of medication (including drugs) which can affect normal brain function, from participation. All participants gave informed consent and were compensated with either 150 euros or a study-participation certificate for course credits (9 h).

### Performance tasks

#### Procedural arithmetic learning task

The arithmetic learning task consisted of a procedural learning and a fact learning part. The latter is analyzed in depth in an ongoing work on arithmetic learning and only mentioned for completeness. For procedural training, participants were presented with a problem in the form A # B. The problems were to be solved by 2 * B–A + 3, hence 4 # 37 = 71, as 2 * 37 = 74 74–6 = 68 68 + 3 = 71. Over the course of the 5 training days, participants were presented with a total of 300 trials. These consisted of 168 (56 on day 1 and 28 on days 2–5) procedural training problems and 4 fact learning problems (appearing once on day 1 and 8 times each on days 2–5). Each procedural trial started with a fixation cross appearing on the screen for 1 s, after which the problem was presented for 8 s and participants were instructed to press a button whenever they have finished calculating. Following that, three options were presented on the screen and participants were instructed to select their solution by pressing one of the three respective buttons within 3 s. One of the presented options was the correct option and the other two options called distractors were randomly generated. Feedback was presented after each trial to tell the participants if they chose the right option or not.

#### Working memory task

The N-back task is a frequently used working memory task in which participants have to determine if the current trial has been presented. The ‘N’ refers to the position of the previous trial that participants have to remember and actively update with every trial. In our study, we used a 2-back task with letters i.e., the target letter was two trials before the current trial. A total of 120 trials were presented, each lasting for 2 s, and the whole task took 4 min in total. In each trial, a letter appeared on the screen for 0.5 s, followed by a black screen appeared for the remaining 1.5 s. Participants had to decide, whether the letter just presented was the same as 2 trials before. If they thought it was the same letter, they were asked to press a corresponding button, and if not, no action was required. They could react during the 2 s of trial duration, in which participants had to make a response. Out of 120 trials, 40 were matching target trials. The N-back task was performed on Day 1 and after the 3 days of stimulation training on Day 5 of the experiment, each time before the arithmetic task and MRI assessment took place.

#### Proactive interference task

The recent probes task was used to assess proactive interference (Monsell 1978). Each trial consisted of a set of four letters (presented for 1 s), and a probe letter appearing shortly (2 s) after. Participants were instructed to react as fast as possible and to press a button if the probe letter was one of the letters from the previous trial set (target letter) and press another button if it was not. Thereby, probe letters could be letters included the trial set and the one previous (recent positive), or a letter included the trial set but not the one previous (non-recent positive), or a letter not included in the trial set but the one previous (recent negative), or a letter neither included in the trial set nor in the previous set (non-recent negative). The whole task consisted of 4 blocks of 24 trials each and lasted for approximately 12 min. The main aspects of interest in this task are the accuracy and the reaction time differences between recent negative and non-recent negative trials as these reflect proactive interference.

### Functional magnetic resonance imaging

#### Data acquisition

MRI scanning was performed using a 3 T Siemens Magnetom Vida MR scanner. Blood Oxygen Level-Dependent (BOLD) fMRI images were acquired using a gradient echo-planar-imaging (EPI) sequence [time of repletion (TR) = 1200 ms, time of echo (TE) = 30 ms, flip angle = 72°, slices/volume = 56, slice thickness = 2.5 mm, multiband acceleration factor = 3]. The anatomical, T1-weighted, images were collected with an ultrafast spoiled gradient echo pulse sequence (T1-TFE; TR = 1.60 ms, TE = 2.38 ms, flip angle = 9°, slices = 224, slice thickness = 1 mm). DWI data were collected using the same 3 T scanner with TR = 2800 ms, TE = 95 ms, flip angle = 90°, slices = 57, and slice thickness = 2.5 mm.

#### Grey matter analysis

Preprocessing was performed using statistical parametric mapping (SPM) 12 (https://www.fil.ion.ucl.ac.uk/spm/), Data Processing and Analysis of Brain Imaging (DPABI) (Yan et al. 2016), and the CONN toolbox (Whitfield-Gabrieli and Nieto-Castanon 2012). Anatomical images were segmented into white matter, gray matter, and cerebrospinal fluid (CSF). Standard preprocessing steps were performed, including slice time correction, motion correction, co-registration with the anatomical image, and outlier identification based on the global signal and frame-wise displacement. Functional images were normalized to standard Montreal Neurological Institute (MNI) space, and spatial smoothing was performed with a 6 mm isotropic Fixed Width Half-Maximum (FWHM) Gaussian Kernel. The data were filtered with a 0.01–0.10 Hz temporal band-pass filter and regressed on motion parameters, white matter, and CSF. For multiple error correction, false discovery rate (FDR) corrections were used (Nichols and Hayasaka 2003) with a voxel threshold of *p* < 0.001 and a cluster threshold of *p* < 0.05.

##### Resting-state network analysis

Resting-state network (RSN) analyses were restricted to the RSN templates provided by the CONN toolbox. Three networks were included: the FPN, the DMN, and the salience network (Keeser et al. 2011; Chand and Dhamala 2016). More detailed information about the included brain regions and networks is provided in Table 1. Statistical analyses were conducted using the CONN 2nd-level analysis module and the SPSS 27 statistical software package (IBM SPSS Statistics, NY, US). To study stimulation-induced changes, within-network connectivity, between-networks connectivity, and network connectivity with the rest of the brain were estimated and compared between groups. Analyses of variance (ANOVA) was conducted for connectivity values with group (stimulation vs. sham) as a between-subjects variable and time (pre- vs. post-stimulation) as a within-subject variable. To correct for multiple error, we used false discovery rate (FDR) corrections. For the significant interactions, we extracted the individual connectivity values within each significant connection separately for each subject, and performed paired samples *t* tests over time in each group. All comparisons were subjected to Bonferroni correction.

**Table 1:** The table shows the seed regions for each of the networks, including the frontoparietal network (FPN), default mode network (DMN), and salience network

RSNs	Region	Coordinates (x, y, z)	Abbreviation
FPN	Left-lateral prefrontal cortex	[− 43 + 33 + 28]	L-LPFC
	Left-posterior parietal cortex	[− 46 − 58 + 49]	L-PPC
	Right-lateral prefrontal cortex	[+ 41 + 38 + 30]	R-LPFC
	Right-posterior prefrontal cortex	[52 − 52 + 45]	R-PPC
DMN	Medial prefrontal cortex	[1 + 55 − 3]	MPFC
	Left-lateral parietal cortex	[− 39 − 77 + 33]	L-LP
	Right-lateral parietal cortex	[+ 47 − 67 + 29]	R-LP
	Posterior cingulate cortex	[+ 1 − 61 + 38]	PPC
Salience network	Anterior cingulate cortex	[0 + 22 + 35]	ACC
	Left-anterior insula	[− 44 + 13 + 1]	L-AI
	Right-anterior insula	[+ 47 + 14 0]	R-AI
	Left-rostral prefrontal cortex	[− 32 + 45 + 27]	L-RPFC
	Right-rostral prefrontal cortex	[+ 32 + 46 + 27]	R-RPFC
	Left-supramarginal gyrus	[− 60 − 39 + 31]	L-SMG
	Right-supramarginal gyrus	[+ 62 − 35 + 32]	R-SMG

##### Seed-based analysis

In addition to the network approach, we defined the Left-Lateral Prefrontal Cortex (L-LPFC) as a region of interest (ROI) to investigate stimulation effects at the stimulation site. Functional connectivity was estimated between this ROI and the whole brain. For statistical analyses, Pearson correlations were used to estimate the strength of functional associations, and z-transformed (Fisher’s *z*). Similar to network-based analysis, FDR correction was applied to account for multiple errors.

#### White matter microstructure analysis

The microstructure of white matter was investigated using Fractional Anisotropy (FA), which is the most commonly used metric in DTI analysis (Lebel and Deoni 2018). Pipeline for Analyzing Brain Diffusion Images (PANDA) was used for preprocessing and analysis (Cui et al. 2013). The data preprocessing steps included converting DICOM files into NIfTI images, estimating the brain mask, removing non-brain tissue, correcting for eddy current and head motion, adjusting the diffusion gradient direction, and then calculating DTI metrics. Once the preprocessing was completed, the individual FA images were registered to the FA standard template in the MNI space using a non-linear method. Atlas-based analysis was employed by PANDA to calculate the regional FA by averaging the values within each region of the John Hopkins University (JHU) White Matter Tractography Atlas (Hua et al. 2008). The FA of three tracts was estimated in the left hemisphere that connects frontal brain regions with other brain areas, including the left inferior fronto-occipital fascicle (L-IFOF), left superior longitudinal fasciculus (L-SLF), and left anterior thalamic radiation (L-ATR). The selection of tracts was based on the study investigating the changes in the architecture of white matter tracts with expertise in mathematics (Jeon et al. 2019) and a review study investigating the changes in the brain activity with arithmetic training (Peters and De Smedt 2018).

### Stimulation parameters

Theta tACS stimulation during the arithmetic training was delivered for 25 min using a battery-driven NeuroConn (NeuroConn, Germany) DC-stimulator Plus. The target electrode was a 3 × 3 cm rubber electrode placed at the F3 location (based on the 10–20 system) targeting the L-DLPFC. The return electrode was a 7 × 5 cm rubber electrode placed on the left shoulder. We also adjusted the stimulation intensity and the simulation frequency for each subject. Stimulation intensity was adjusted with a stepwise increase of 0.25 mA starting at 1 mA until either the participants reported skin sensations or phosphenes or the current reached 1.5 mA. If skin sensations or phosphenes were reported at or below 1 mA, the current intensity was reduced by 0.1 mA until no sensations were reported. We used a similar procedure for the sham group, but the stimulation lasted only 30 s. Theta frequency was determined based on resting-state IAF assessment (IAF minus 5 Hz) before the arithmetic learning session. This was based on the calculation of individual frequency bands proposed by (Klimesch 1999). In this work, theta band was defined in the range of 6–4 Hz below IAF. Hence, IAF minus 5 Hz puts the stimulation frequency in the middle of the individual theta band. Participants were asked to close their eyes for 2 min while we recorded EEG data on a limited set of electrodes (P5, P3, P1, Pz, P2, P4, and P6). Using the MNE Toolbox (Gramfort et al. 2013) and a custom-built Python code, we determined the individual stimulation frequency. Similar approaches to individualize stimulation frequency have been used successfully in several prior studies (e.g. Neuling et al. 2013; Kasten et al. 2016; Vosskuhl et al. 2016; Mosbacher et al. 2021). An overview of the mean stimulation intensities and frequencies is given in Table 2.

**Table 2:** The mean and standard deviations of the stimulation intensities and frequencies assessed for the different groups on the three stimulation days are shown

Stimulation	Day	Intensity		Frequency	
		M	SD	M	SD
Theta band tACS	1	1.08	0.31	5.28	1.07
	2	0.97	0.27	5.12	1.18
	3	1.05	0.26	5.28	1.15
Sham	1	1.08	0.25	4.82	1.35
	2	1.13	0.27	4.66	0.89
	3	1.10	0.26	4.92	1.39

## Results

### Performance tasks

The ANOVA test conducted on performance in procedural calculation showed no significant interaction in accuracy (*F* = 2.04, *p* = 0.159) or reaction time (*F* = 0.01, *p* = 0.943) between the two groups on Day 5 compared to Day 1. However, the main effect of time was observed for procedural accuracy (*F* = 19.26, *p* < 0.001) and reaction time (*F* = 517.64, *p* < 0.001). Participants exhibited better performance on Day 5 compared to Day 1, as indicated by a higher accuracy percentage (*M* = 81.93, Sd = 12.40) versus (*M* = 72.61, Sd = 15.75) and faster calculation times to trials (*M* = 2.59, Sd = 0.77) versus (*M* = 5.18, Sd = 0.76) on Day 5 and Day 1, respectively. In terms of working memory, there was no significant interaction for accuracy (*F* = 0.28, *p* = 0.603) or reaction time (*F* = 1.23, *p* = 0.273). The main effect of time was only significant for accuracy (*F* = 34.182, *p* < 0.001) and not for reaction time (*F* = 0.03, *p* = 0.872). Participants were more accurate on Day 5 (*M* = 83.44, Sd = 13.06) compared to Day 1 (*M* = 75.89, Sd = 14.76). Furthermore, the proactive interference task did not show any significant main effects for accuracy (*F* = 0.18, *p* = 0.673) or reaction time (*F* = 0.99, *p* = 0.323), and there was no interaction observed for either of the measures, i.e., accuracy (*F* = 2.09, *p* = 0.155) and reaction time (*F* = 0.69, *p* = 0.410).

### Modulation of frontoparietal network connectivity

**Fig. 2:**
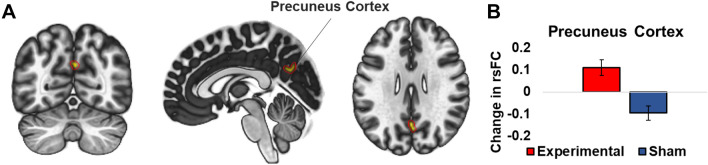
Changes in functional connectivity for FPN. (**A**) shows connectivity changes between the FPN network and a cluster in the precuneus cortex after stimulation training. (**B**) shows the follow-up analysis. An increase in resting state functional connectivity in the stimulation group, while a decrease in the sham group was observed. The error bars indicate the standard deviation

ANOVA revealed a significant interaction between the frontoparietal network (FPN) and the precuneus cortex [*F* (4, 192) = 4.82, p-FDR-corrected < 0.001] as shown in Fig. 2A. Further analysis revealed a significant increase in functional connectivity in the stimulation group [*t* (24) = 3.10, *p* = 0.005, *d* = 0.623] and a decrease in the sham group [*t* (24) =  − 2.93, *p* = 0.007, *d* = 0.585] depicted in Fig. 2B. The outcomes are described in more detail in Table 3, and there were no other significant main or interaction effects. Additionally, the observed change in connectivity did not correlate with any behavioral improvements observed in either the experimental or sham groups.

**Table 3:** The significant clusters identified after RSN analysis and seed-based analysis are shown

Clusters	Cluster (x, y, z)	Cluster size	p-FDR	Regions
**FPN** Cluster 1	[+ 02 − 66 + 28]	52 voxels	0.000033	Precuneus cortex
**L-LPFC** Cluster 1	[− 04 − 64 + 32]	114 voxels	0.000011	Precuneus cortex
Cluster 2	[− 02 − 46 + 10]	98 voxels	0.000021	Cingulate gyrus, posterior division
Cluster 3	[− 40 − 72 + 36]	65 voxels	0.001761	Lateral occipital cortex, superior division left

For RSN analysis, a significant cluster was identified in the precuneus cortex with FPN. With L-LPFC selected as a seed, three clusters were identified, including precuneus cortex, cingulate gyrus, and lateral occipital cortex. P-FDR represents the *p* value after false discovery rate correction. Coordinates are reported in MNI space

### Modulation of connectivity with parietal and occipital brain regions

**Fig. 3:**
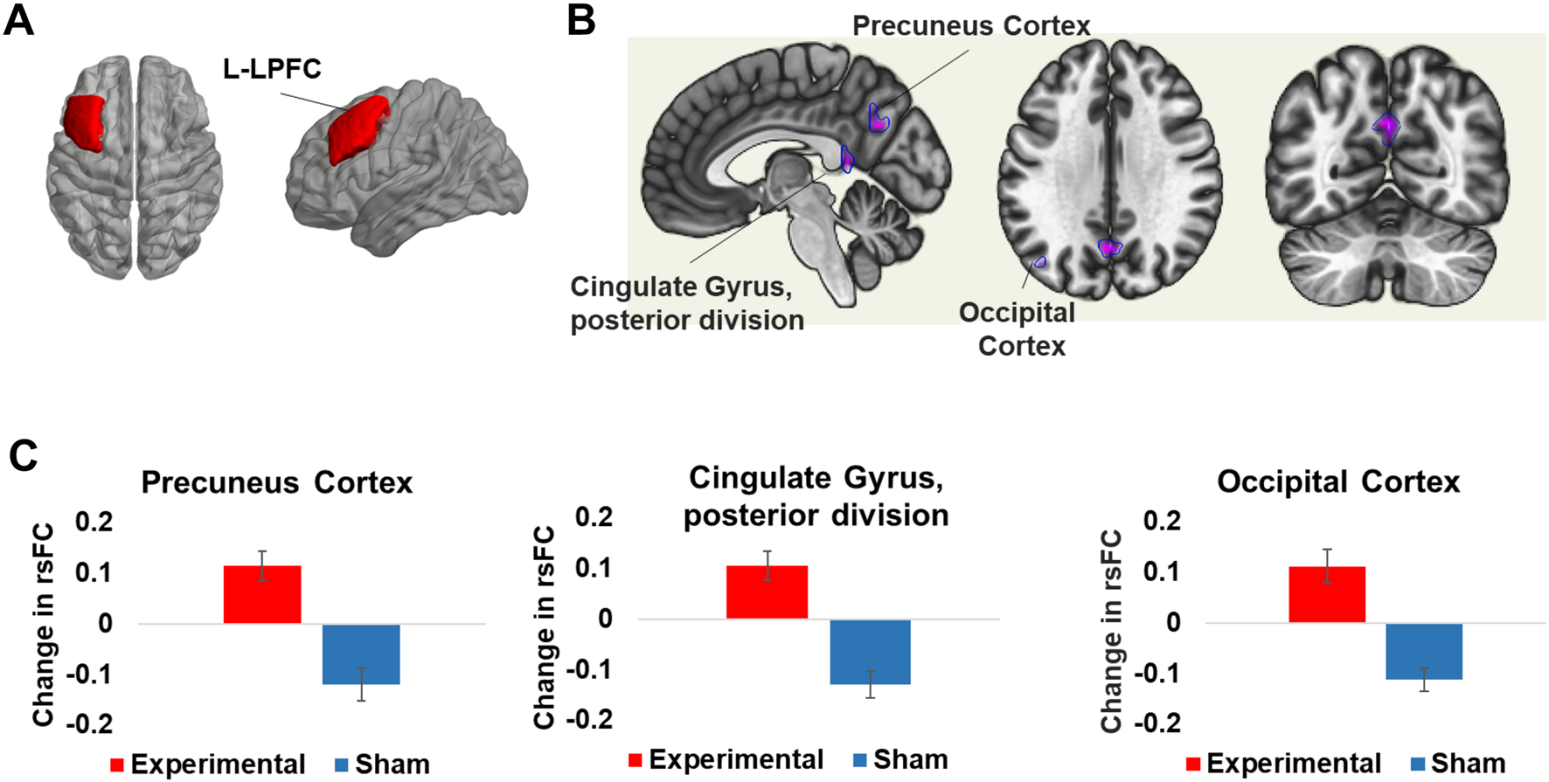
Seed-based connectivity analysis was performed by defining a seed at the stimulated brain region as shown in (**A**). (**B**) shows the significant clusters, including Precuneus Cortex, Cingulate Gyrus, posterior division, and Occipital Cortex. (**C**) shows the change in connectivity for the identified clusters in the experimental and sham groups. An increase in connectivity was observed in the experimental group, while a decrease was observed in the sham group for all three clusters. The error bars indicate the standard deviation

With seed selected at the stimulated brain region as shown in Fig. 3A, a significant Group × Time interaction was observed, and three clusters were identified: the precuneus (p-FDR-corrected < 0.001), the PCC (p-FDR-corrected < 0.001), and the left occipital cortex (p-FDR-corrected < 0.001) as shown in Fig. 3B and Table 1. Further analysis indicated that the stimulation group had increased connectivity for all three clusters i.e., precuneus [*t*(24) = 3.95, *p* = 0.001, *d* = 0.790], PCC [*t*(24) = 3.72, *p* = 0.001, *d* = 0.744], and left occipital cortex [*t*(24) = 3.41, *p* = 0.002, *d* = 0.682], while the sham group showed a decrease in connectivity for all three clusters i.e., precuneus [*t*(24) = 3.70, *p* = 0.001, *d* = 0.740], PCC [*t*(24) = 4.86, *p* < 0.001, *d* = 0.834], and left occipital cortex [*t*(24) = 4.90, *p* < 0.001, *d* = 0.981] as presented in Fig. 3C. None of these changes correlated with changes in performance markers for experimental and sham groups.

### Increase in fractional anisotropy (FA) in both groups after training

**Fig. 4:**
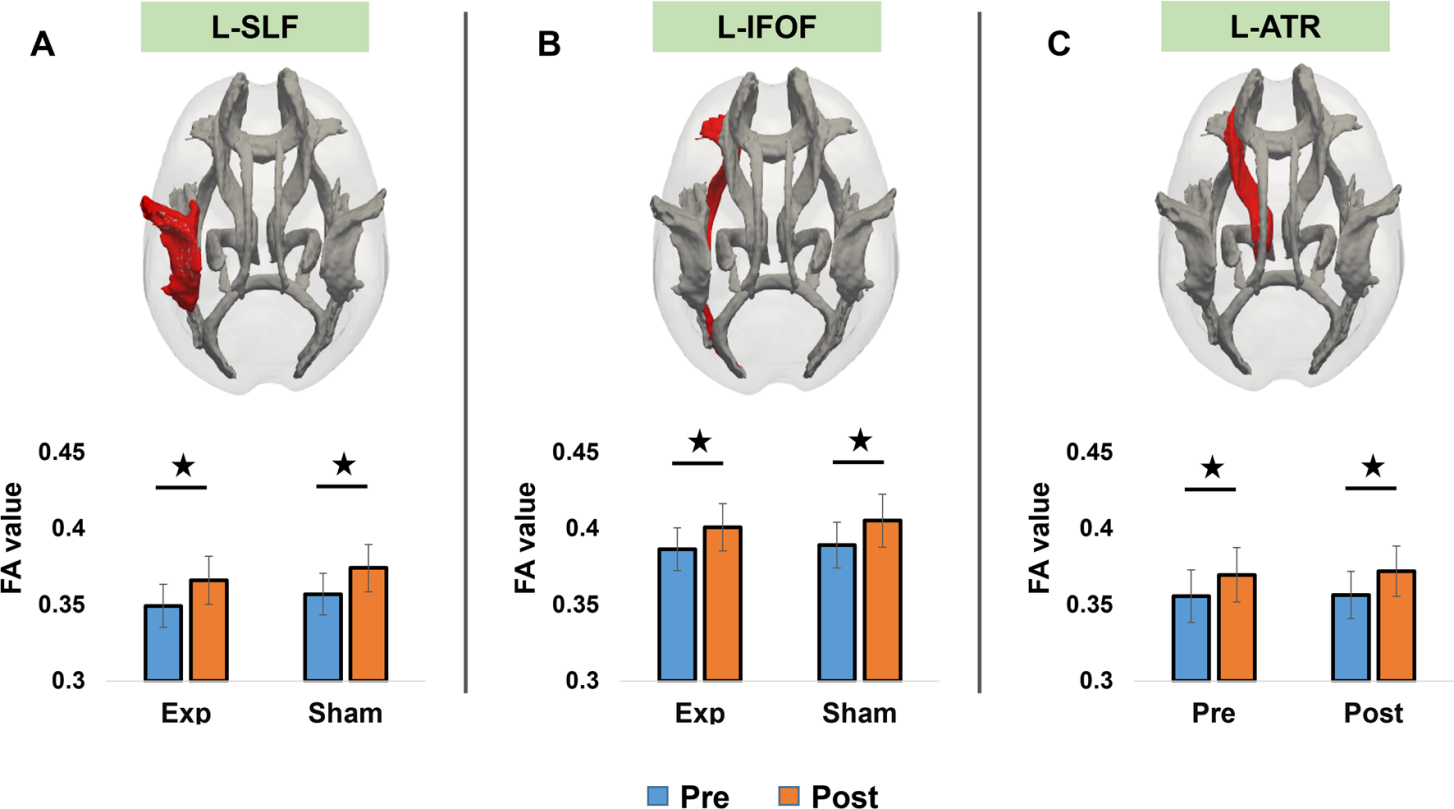
The study observed an increase in FA values for the white matter tracts L-SLF (**A**), L-IFOF **(B**), and L-ATR (**C**) in both the experimental and sham groups. However, there were no significant differences found in the FA values between the two groups for the selected tracts when analyzing their interaction. The error bars represent standard deviation

There was no significant interaction for FA values between the experimental and sham groups for the selected tracts, namely SLF (*F* = 0.03, *p* = 0.859), IFOF (*F* = 0.59, *p* = 0.447) and ATR (*F* = 0.30, *p* 0.585). However, a significant main effect for time was observed for SLF (*F* = 312.53, *p* < 0.001), IFOF (*F* = 211.96, *p* < 0.001), and ATR (*F* = 254.20, *p* < 0.001), FA values increased in both groups on Day 5 as compared to Day 1 as shown in Fig. 4. Further exploratory analysis using Spearman correlation showed that the changes in these tracts were also correlated with performance measures in arithmetic training. For example, the improvement in accuracy for arithmetic training (difference between Day 5 and Day 1) was moderately correlated with the increase in FA values for IFOF (*r* = 0.39, *p* = 0.050) for the experimental group and increase in FA values for IFOF (*r* = 0.40, *p* = 0.045) in the sham group.

## Discussion

The entrainment of ongoing oscillatory activity using brain stimulation opens an exciting possibility to drive neural changes in the brain circuitry and eventually modulate behavioral performance. For example, theta band stimulation of the L-DLPFC region has been reported to enhance arithmetic learning (Mosbacher et al. 2021), and it has also been reported to induce changes in the resting-state functional brain activity (Abellaneda-Perez et al. 2019). These stimulation-related effects have mostly been observed in single-session studies; however, it is not clear if the effect occurs for repetitive stimulation during cognitive training over multiple days. The current study investigated if repeated stimulation over the L-DLPFC region during arithmetic learning could induce changes in resting-state functional connectivity and white matter structural integrity. Participants underwent 3 days (Day 2 to Day 4) of arithmetic training along with stimulation. On both Day 1 and Day 5 of the experiment, data were gathered through resting-state fMRI, DWI, and behavioral assessments. The results demonstrated that stimulation of the L-DLPFC induces significant changes in the connectivity of the FPN network linking the L-DLPFC with regions in the parietal cortex. However, no significant differences were observed in white matter networks after stimulation.

Analysis of the grey matter networks showed a significant increase in connectivity of the FPN with the precuneus in the stimulation group and a decrease in the sham group. A seed-based analysis further showed that connectivity changes of the precuneus were associated with the stimulated brain region. The precuneus, situated in the posterior part of the medial parietal cortex, is a crucial brain region involved in various complex cognitive tasks with arithmetic being among them (Cavanna and Trimble 2006; Fehr et al. 2007). A remarkable study by Utevsky and colleagues (Utevsky et al. 2014) compared functional changes in the brain when participants were engaged in reward-based decision tasks requiring externally focused attention as compared to rest. They observed state-dependent functional connectivity in the precuneus in a way that the functional connectivity between FPN and precuneus increased during the task, while the connectivity between DMN and precuneus increased during the resting state. The authors concluded that the precuneus interacts with DMN and FPN to distinguish cognitive states. In our study, stimulation selectively increased the functional connectivity between precuneus and FPN after training. Moreover, the modulation of FPN holds clinical significance, as evidenced by various studies. For example, a meta-analysis of resting-state functional connectivity in Major depressive disorder (MDD) reported hypo-connectivity within FPN (Kaiser et al. 2015). Similarly, disruption of the FPN has also been observed in schizophrenia (Chahine et al. 2017), spatial neglect (He et al. 2007), and bipolar disorder (Rai et al. 2021). These findings suggest that future investigations could potentially explore the therapeutic potential of modulating the FPN in neuropsychiatric patients.

Along with the precuneus, we observed significant connectivity changes between L-LPFC and PCC. PCC is a critical node in the DMN which lies inferior to precuneus cortex in the medial parietal cortex and forms a crucial region actively engaged in various cognitive tasks (Leech and Sharp 2014). Although, there is no consensus on a specific role of PCC, a widely accepted hypothesis proposes that the PCC plays a pivotal role in facilitating internally directed cognition, based on evidence that PCC activity increases during retrieval of autobiographical memories (Gusnard et al. 2001), future planning (Addis et al. 2007), and mind wandering during resting state (Mason et al. 2007). In addition, the interaction of PCC and precuneus with other brain regions has been reported to represent conscious awareness (Vogt and Laureys 2005). Our study did not aim to investigate internally directed cognition or conscious awareness. However, modulation of PCC by stimulation of L-DLPFC suggests that this stimulation protocol may have the potential to impact the aforementioned cognitive functions. Two recent studies stimulating the L-DLPFC with tACS reported modulation of parietal brain regions after one stimulation session (Abellaneda-Perez et al. 2019; Mondino et al. 2019). In the first study, tACS decreased the functional connectivity during rest while the connectivity increased during a working memory task. The study by Mondino and colleagues (Mondino et al. 2019) reported that tACS increased functional connectivity between L-DLPFC and parietal brain regions. The modulated region reported in this study is more lateralized and cortical than the area reported in our study, possibly due to differences in stimulation parameters or montage. Despite some inconsistency in the direction of change induced by stimulation, the available evidence suggests that stimulating the L-DLPFC affects the connectivity between frontoparietal brain regions.

Furthermore, there was a significant difference in connectivity identified with the occipital cortex, which is primarily accountable for visual processing. Generally speaking DLPFC and occipital cortices are considered functionally disconnected. However, findings from fMRI studies have indicated that during working memory tasks, the DLPFC exhibits distinct top-down control over the parietal or occipital cortices (Feredoes et al. 2011; Zanto et al. 2011; Kundu et al. 2015). It could be that the observed effect is due to indirect connections between the two regions, perhaps via other intermediate brain regions. In addition, several other studies have reported that tACS can produce phosphines in the visual cortices (Lazarev et al. 2004; Schwiedrzik 2009; Mencarelli et al. 2022) and the intensity of stimulation in our study was determined by the participants' reports of phosphines, indicating that this factor may have contributed to the modulation of occipital region. However, additional investigation is necessary to establish the precise mechanisms that account for this phenomenon.

Another interesting finding in our study was the decrease in functional connectivity in the sham group following arithmetic training. It might seem surprising at first, but several research studies have demonstrated that resting-state functional connectivity is not an absolute brain state and can be modulated by the task. For example, the study by Lewis and colleagues (Lewis et al. 2009) demonstrated that after a visual perceptual learning task, the connectivity in the visual network and fronto-parietal brain areas was significantly modulated. This sort of modulation in resting-state functional connectivity has also been observed in hippocampal-cortical areas after participants engaged in an episodic memory task (Tambini et al. 2010) and language processing areas after a language task (Waites et al. 2005). In addition, previous studies targeting different areas of the brain have reported such opposing effects in fMRI connectivity between experimental and sham groups (Abellaneda-Perez et al. 2019; Mondino et al. 2019; Khan et al. 2020) which supports our results.

To explore changes in white matter microstructure in the left prefrontal region, we conducted FA analysis. Specifically, we used the JHU WM tractography atlas to investigate the SLF, IFOF, and ATR fiber tracts. Following arithmetic training, both groups exhibited increased FA values in the left SLF, ILF, and ATR. Furthermore, improvements in accuracy on the procedural arithmetic learning task were positively correlated with changes in the IFOF and ATR fiber tracts. Previous research has established a clear connection between the integrity of white matter networks and arithmetic performance (Peters and De Smedt 2018; Jeon et al. 2019). Our study builds on this literature by directly linking the increase in FA values to improved performance on the procedural learning task. Here, it is worth noting that while we observed an interaction effect on resting state gray matter connectivity, there was no corresponding effect on structural integrity which possibly indicates that stimulation resulted in connectivity of brain regions but it did not alter the underlying physical properties of the white matter tracts connecting them.

The primary goal of manipulating brain activity by stimulation is to induce a change in behavior that can help us draw causal inferences about the functional role of the stimulated region. However, behavioral results showed an overall improvement on Day 5 as compared to Day 1 but did not show any significant difference in the experimental group compared to the sham group. One possible explanation for observing an effect in functional connectivity of the brain and not in behavior could be that the cognitive tasks used in these analyses might not be sensitive enough to detect subtle changes in brain function or behavior. This inconsistency is not new in the field of non-invasive brain stimulation. For example, the study by Abellaneda and colleagues, which showed changes in the precuneus cortex and PCC after single session tACS on L-DLPFC, did not observe any improvement in working memory performance in healthy individuals (Abellaneda-Perez et al. 2019). The study by Mondino et al. also did not report any behavioral modulation after one-session stimulation (Mondino et al. 2019). However, some other studies with slightly different montages have improved behavioral performance. For example, bilateral tACS stimulation over DLPFC improved performance in verbal working memory task (Meiron and Lavidor 2014). Another study utilizing cross-frequency protocols with theta-gamma synchronization enhanced performance in spatial working memory task (Alekseichuk et al. 2016). In addition, a recent study examined the effects of multiple session theta and gamma stimulation of frontal and parietal brain regions on working memory and long-term memory (Grover et al. 2022). The study found that theta stimulation of L-DLPFC did not affect primacy or recency effects in a free recall memory task. However, gamma stimulation of L-DLPFC and theta stimulation of the parietal cortex resulted in improvements in primacy and recency, respectively. Overall, alterations in connectivity may not always translate to changes in behavioral performance, and a number of studies have documented performance changes with slightly different stimulation protocols. Future studies should conduct a more methodical exploration of different protocols to understand this inconsistency.

This study has several limitations. First, we utilized a conventional stimulation montage with large electrode size, which targeted a broad area of the brain, thereby lacking specificity in the stimulation and potentially affecting non-targeted brain regions. This lack of specificity makes it challenging to ascertain the exact function of the target brain region in a particular task. More targeted stimulation methods, such as high definition transcranial electrical stimulation (Bikson et al. 2019) and temporal interference stimulation (Grossman et al. 2017) are available. Additionally, although we personalized the stimulation intensity and frequency, the location of the stimulation was not individualized. Future studies using more focused stimulation methods should incorporate structural and functional scans to accurately localize the target region and ensure precise stimulation of intended brain region. Furthermore, our study did not include an anatomical control group, meaning there was no other stimulation group with active stimulation on a non-target region such as the vertex, to control for the effects associated with the sensation of stimulation. Future studies should incorporate these aspects in the study design.

In conclusion, our study provides evidence that multiple session theta-band stimulation of L-DLPFC modulates interconnected brain regions in the parietal and occipital cortices. However, the structural integrity of white matter regions and behavioral measures were uninfluenced by stimulation.

## Data Availability

The datasets generated during and/or analyzed during the current study are available from the corresponding author on reasonable request.
